# Potassium clavulanate

**DOI:** 10.1107/S1600536810027984

**Published:** 2010-07-21

**Authors:** Kotaro Fujii, Kazuyuki Toyota, Akiko Sekine, Hidehiro Uekusa, Ilma Nugrahani, Sukmadjaja Asyarie, N. Sundani Soewandhi, Slamet Ibrahim

**Affiliations:** a Department of Chemistry and Materials Science, Tokyo Institute of Technology, Ookayama, Meguro, Tokyo 152-8551, Japan; b School of Pharmacy, Institut Teknologi Bandung, Ganesha 10, Bandung, 40312, Indonesia

## Abstract

The title salt, K^+^·C_8_H_8_NO_5_^−^ [systematic name: potassium (2*R*,5*R*,*Z*)-3-(2-hy­droxy­ethyl­idene)-7-oxo-4-oxa-1-aza­bicyclo­[3.2.0]heptane-2-carb­oxyl­ate], a widely used β-lactam anti­biotic, is usually chemically unstable even in the solid state owing to its tendency to be hydrolysed. In the crystal structure, the potassium cations are arranged along the *a* axis, forming inter­actions to the carboxyl­ate and hy­droxy groups, resulting in one-dimensional ionic columns. These columns are arranged along the *b* axis, connected by O—H⋯O hydrogen bonds, forming a layer in the *ab* plane.

## Related literature

For the pharmacological activity of clavulanic acid and potassium clavulanate, see: Bird *et al.* (1982 ▶); Mayer & Deckwer (1996 ▶); Navarro (2005 ▶). For the hydrolysis properties of clavulanic acid and potassium clavulanate, see: Bersanetti *et al.* (2005 ▶); Brethauer *et al.* (2008 ▶); Haginaka *et al.* (1985 ▶); Hickey *et al.* (2007 ▶); Saudagar *et al.* (2008 ▶).
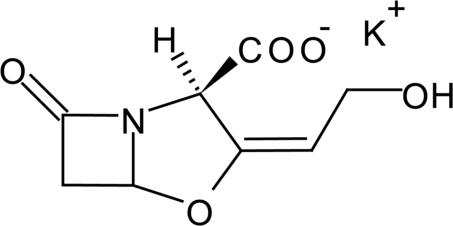


## Experimental

### 

#### Crystal data

K^+^·C_8_H_8_NO_5_^−^
*M* *_r_* = 237.25
Orthorhombic, 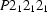

*a* = 4.3453 (6) Å
*b* = 7.8191 (11) Å
*c* = 27.491 (3) Å
*V* = 934.1 (2) Å^3^
*Z* = 4
Mo *K*α radiation
μ = 0.57 mm^−1^
*T* = 173 K
0.24 × 0.04 × 0.01 mm

#### Data collection

Rigaku R-AXIS RAPID IP area-detector diffractometer
Absorption correction: multi-scan (*ABSCOR*; Higashi, 1995 ▶) *T*_min_ = 0.876, *T*_max_ = 0.994
9047 measured reflections
2138 independent reflections
1433 reflections with *I* > 2σ(*I*)
*R*_int_ = 0.088

#### Refinement

*R*[*F* ^2^ > 2σ(*F* ^2^)] = 0.044
*wR*(*F* ^2^) = 0.118
*S* = 1.12
2138 reflections
137 parameters
H-atom parameters constrained
Δρ_max_ = 0.52 e Å^−3^
Δρ_min_ = −0.56 e Å^−3^
Absolute structure: Flack (1983 ▶), 839 Friedel pairs
Flack parameter: −0.05 (9)

### 

Data collection: *PROCESS-AUTO* (Rigaku, 1998 ▶); cell refinement: *PROCESS-AUTO*; data reduction: *PROCESS-AUTO*; program(s) used to solve structure: *SHELXS97* (Sheldrick, 2008 ▶); program(s) used to refine structure: *SHELXL97* (Sheldrick, 2008 ▶); molecular graphics: *ORTEP-3 for Windows* (Farrugia, 1997 ▶); software used to prepare material for publication: *SHELXL97*.

## Supplementary Material

Crystal structure: contains datablocks I, global. DOI: 10.1107/S1600536810027984/tk2689sup1.cif

Structure factors: contains datablocks I. DOI: 10.1107/S1600536810027984/tk2689Isup2.hkl

Additional supplementary materials:  crystallographic information; 3D view; checkCIF report

## Figures and Tables

**Table 1:** Selected bond lengths (Å)

O2—K1^i^	2.773 (3)
O2—K1^ii^	2.799 (3)
O2—K1	2.827 (3)
O3—K1	2.786 (3)
O4—K1^ii^	2.818 (4)
O4—K1^iii^	2.865 (4)

Symmetry codes: (i) 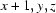; (ii) 
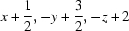
; (iii) 
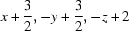
.

**Table 2:** Hydrogen-bond geometry (Å, °)

*D*—H⋯*A*	*D*—H	H⋯*A*	*D*⋯*A*	*D*—H⋯*A*
O4—H4*A*⋯O3^iv^	0.84	1.90	2.673 (5)	153

Symmetry code: (iv) 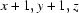.

**Fig. 1.**
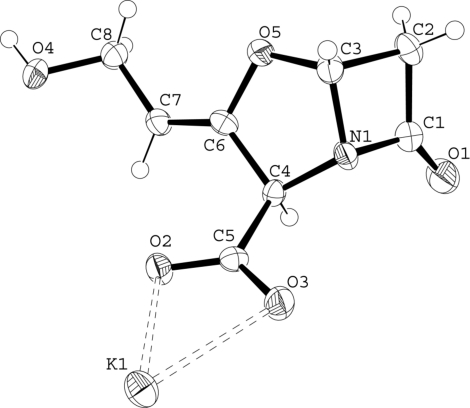
Molecular structure of potassium clavulanate showing numbering scheme and displacement ellipsoids at the 50% probability .

**Fig. 2.**
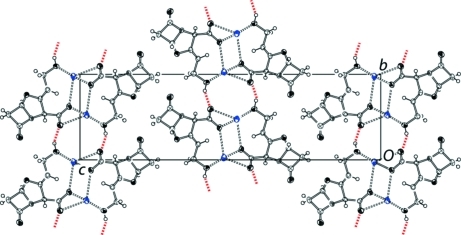
Crystal packing viewed along the a axis. The hydrogen bonds are shown with red dotted lines.

## supplementary crystallographic information

### Comment

Many pathogenic bacteria secrete β-lactamases as a defense mechanism against β-lactam antibiotics. Because such β-lactamases have the potential to inactivate β-lactam antibiotics, inhibitors for these β-lactamases are clinically very important. Clavulanic acid (CA) is a powerful naturally obtained inhibitor for bacterial β-lactamases produced by the organism *Streptomyces clavuligerus*. Although CA itself can act as an β-lactam antibiotic and is active against a wide spectrum of Gram-positive and Gram-negative bacteria (Mayer & Deckwer, 1996), it is much more effective as a drug in combination with β-lactamase-sensitive penicillins, such as amoxicillin. In that situation, CA protects the β-lactam ring of the amoxicillin from hydrolysis and can maintain its activity against β-lactamase producing bacteria (Bird *et al.*, 1982). The CA potassium salt is widely used as a drug in injectable and solid form, especially combined with amoxicillin sodium and amoxicillin trihydrate (Navarro, 2005).

In this context, an understanding of the structure of CA is important in order to establish its ability to form molecular interactions. Unfortunately, CA is chemically unstable as are the other β-lactam antibiotics, being very sensitive to pH, temperature, and humidity *via* the hydrolysis degradation mechanism; (Bersanetti *et al.*, 2005; Hickey *et al.*, 2007; Saudagar *et al.*, 2008). The decomposition is also self-catalyzed (Brethauer *et al.* 2008; Haginaka *et al.* 1985) and there have been some difficulties in obtaining a single crystals of CA. Therefore, until now there has been no report of a crystal structure of CA. In this study, single crystals of potassium clavulanate were successfully obtained by a low-temperature crystallization process and the crystal structure was determined.

In the molecular structure of potassium clavulanate, Fig. 1, the C5–O2 and C5–O3 distances of 1.262 (5)Å and 1.256 (5) Å, respectively, indicate that the negative charge of the carboxylate group is delocalised. The potassium cation is surrounded by six oxygen atoms, three O2, one O3 and two O4, deriv ed from four different clavulanate anions. The selected bond lengths around the potassium cation are listed in Table 1. These interactions are infinitely linked along the a axis and lead to an ionic (hydrophilic) column structure. These columns are connected by intermolecular O–H···O hydrogen bonds formed between O4-hydroxyl groups and carboxylate-O2 atoms, and form a hydrophobic layer in the *ab* plane; Fig. 2. By contrast, the remaining hydrophobic groups (*i.e.* bicyclo groups) form a hydrophobic layer so that the crystal structure comprises alternating hydrophilic and hydrophilic regions.

### Experimental

The single crystals of potassium clavulanate were grown at a low temperature in order to prevent decomposition. After the compound was dissolved into an 8:2 mixture of methanol/water, a few drops of 1-propanol were added to the solution and the solution was kept at 235 K for a few days. The crystal used in the analysis was immediately covered with inert oil in order to prevent the decomposition through contact with atmospheric water vapor.

### Refinement

The O- and C-bound H atoms were geometrically placed (O–H = 0.84 Å and C–H = 0.95–1.00 Å,) and refined as riding with *U_iso_*(H) = 1.2*U_eq_*(carrier atom). The absolute structure was assigned according to the known configuration of the acid, an assignment confirmed by the refinement of the Flack parameter (Flack, 1983).

### Crystal data

**Table:**

K^+^·C_8_H_8_NO_5_^−^	*F*(000) = 488
*M**_r_* = 237.25	*D*_x_ = 1.687 Mg m^−^^3^
Orthorhombic, *P*2_1_2_1_2_1_	Mo *K*α radiation, λ = 0.71075 Å
Hall symbol: P 2ac 2ab	Cell parameters from 9047 reflections
*a* = 4.3453 (6) Å	θ = 3.0–27.5°
*b* = 7.8191 (11) Å	µ = 0.57 mm^−^^1^
*c* = 27.491 (3) Å	*T* = 173 K
*V* = 934.1 (2) Å^3^	Platet, colorless
*Z* = 4	0.24 × 0.04 × 0.01 mm

### Data collection

**Table:**

Rigaku R-AXIS RAPID IP area-detector diffractometer	2138 independent reflections
Radiation source: rotating anode, Rigaku UltraX18	1433 reflections with *I* > 2σ(*I*)
graphite	*R*_int_ = 0.088
ω scan	θ_max_ = 27.5°, θ_min_ = 3.0°
Absorption correction: multi-scan (*ABSCOR*; Higashi, 1995)	*h* = −5→5
*T*_min_ = 0.876, *T*_max_ = 0.994	*k* = −10→10
9047 measured reflections	*l* = −35→35

### Refinement

**Table:**

Refinement on *F*^2^	Secondary atom site location: difference Fourier map
Least-squares matrix: full	Hydrogen site location: inferred from neighbouring sites
*R*[*F*^2^ > 2σ(*F*^2^)] = 0.044	H-atom parameters constrained
*wR*(*F*^2^) = 0.118	*w* = 1/[σ^2^(*F*_o_^2^) + (0.0305*P*)^2^ + 0.8726*P*] where *P* = (*F*_o_^2^ + 2*F*_c_^2^)/3
*S* = 1.12	(Δ/σ)_max_ = 0.001
2138 reflections	Δρ_max_ = 0.52 e Å^−^^3^
137 parameters	Δρ_min_ = −0.56 e Å^−^^3^
0 restraints	Absolute structure: Flack (1983), 839 Friedel pairs
Primary atom site location: structure-invariant direct methods	Flack parameter: −0.05 (9)

### Special details

**Table:**

Geometry. All e.s.d.'s (except the e.s.d. in the dihedral angle between two l.s. planes) are estimated using the full covariance matrix. The cell e.s.d.'s are taken into account individually in the estimation of e.s.d.'s in distances, angles and torsion angles; correlations between e.s.d.'s in cell parameters are only used when they are defined by crystal symmetry. An approximate (isotropic) treatment of cell e.s.d.'s is used for estimating e.s.d.'s involving l.s. planes.
Refinement. Refinement of *F*^2^ against ALL reflections. The weighted *R*-factor *wR* and goodness of fit *S* are based on *F*^2^, conventional *R*-factors *R* are based on *F*, with *F* set to zero for negative *F*^2^. The threshold expression of *F*^2^ > σ(*F*^2^) is used only for calculating *R*-factors(gt) *etc*. and is not relevant to the choice of reflections for refinement. *R*-factors based on *F*^2^ are statistically about twice as large as those based on *F*, and *R*- factors based on ALL data will be even larger.

### Fractional atomic coordinates and isotropic or equivalent isotropic displacement parameters (Å^2^)

**Table:**

	*x*	*y*	*z*	*U*_iso_*/*U*_eq_	
C1	1.2101 (11)	0.3967 (6)	1.20066 (15)	0.0297 (11)	
C2	1.1168 (11)	0.5309 (6)	1.23813 (13)	0.0276 (9)	
H2A	1.2901	0.5798	1.2569	0.033*	
H2B	0.9447	0.4964	1.2596	0.033*	
C3	1.0199 (11)	0.6369 (6)	1.19340 (14)	0.0250 (10)	
H3	0.8027	0.6790	1.1942	0.030*	
C4	1.2417 (9)	0.5363 (6)	1.11851 (13)	0.0192 (8)	
H4	1.4273	0.4611	1.1163	0.023*	
C5	1.0676 (9)	0.5213 (6)	1.06982 (13)	0.0201 (8)	
C6	1.3526 (11)	0.7148 (5)	1.13197 (12)	0.0218 (9)	
C7	1.5398 (10)	0.8175 (6)	1.10754 (14)	0.0245 (10)	
H7	1.6169	0.7752	1.0775	0.029*	
C8	1.6419 (11)	0.9914 (5)	1.12198 (13)	0.0269 (9)	
H8A	1.5056	1.0382	1.1476	0.032*	
H8B	1.8551	0.9881	1.1346	0.032*	
O1	1.3545 (10)	0.2661 (4)	1.20053 (11)	0.0458 (9)	
O2	1.1415 (8)	0.6217 (4)	1.03630 (9)	0.0258 (7)	
O3	0.8682 (9)	0.4058 (4)	1.06647 (10)	0.0318 (7)	
O4	1.6262 (9)	1.0947 (4)	1.07905 (10)	0.0321 (8)	
H4A	1.6958	1.1926	1.0851	0.038*	
O5	1.2401 (7)	0.7604 (4)	1.17783 (9)	0.0266 (7)	
N1	1.0715 (8)	0.4860 (5)	1.16197 (10)	0.0242 (8)	
K1	0.6334 (2)	0.52404 (13)	0.97836 (3)	0.0284 (2)	

### Atomic displacement parameters (Å^2^)

**Table:**

	*U*^11^	*U*^22^	*U*^33^	*U*^12^	*U*^13^	*U*^23^
C1	0.036 (3)	0.027 (2)	0.026 (2)	−0.001 (2)	0.001 (2)	0.0026 (19)
C2	0.029 (2)	0.035 (2)	0.0185 (16)	−0.001 (3)	−0.0026 (18)	0.0049 (18)
C3	0.026 (2)	0.025 (2)	0.024 (2)	0.0001 (19)	0.0021 (19)	0.0033 (18)
C4	0.0183 (18)	0.018 (2)	0.0211 (17)	−0.0025 (18)	−0.0009 (15)	0.0023 (17)
C5	0.017 (2)	0.022 (2)	0.0214 (17)	−0.0007 (18)	0.0031 (15)	−0.0040 (17)
C6	0.020 (2)	0.028 (2)	0.0174 (17)	0.001 (2)	−0.0018 (18)	−0.0019 (16)
C7	0.027 (2)	0.026 (2)	0.0204 (18)	−0.0043 (19)	0.0037 (18)	0.0005 (17)
C8	0.030 (2)	0.025 (2)	0.0255 (18)	−0.003 (2)	−0.002 (2)	0.0008 (18)
O1	0.070 (3)	0.033 (2)	0.0340 (16)	0.020 (2)	0.006 (2)	0.0063 (15)
O2	0.0265 (16)	0.0296 (17)	0.0213 (12)	−0.0041 (16)	−0.0002 (13)	0.0045 (12)
O3	0.0366 (19)	0.0304 (17)	0.0285 (14)	−0.0127 (16)	−0.0071 (15)	0.0038 (13)
O4	0.046 (2)	0.0201 (15)	0.0304 (15)	−0.0063 (17)	−0.0020 (17)	0.0034 (12)
O5	0.0328 (18)	0.0248 (17)	0.0221 (13)	−0.0045 (13)	0.0064 (13)	−0.0013 (13)
N1	0.032 (2)	0.0236 (19)	0.0174 (14)	0.0030 (17)	0.0036 (14)	0.0043 (14)
K1	0.0242 (4)	0.0362 (5)	0.0248 (4)	0.0008 (5)	−0.0007 (4)	0.0031 (4)

### Geometric parameters (Å, °)

**Table:**

C1—O1	1.199 (6)	C7—H7	0.9500
C1—N1	1.408 (5)	C8—O4	1.432 (5)
C1—C2	1.525 (6)	C8—H8A	0.9900
C2—C3	1.542 (5)	C8—H8B	0.9900
C2—H2A	0.9900	O4—H4A	0.8400
C2—H2B	0.9900	O2—K1^i^	2.773 (3)
C3—O5	1.425 (5)	O2—K1^ii^	2.799 (3)
C3—N1	1.480 (5)	O2—K1	2.827 (3)
C3—H3	1.0000	O3—K1	2.786 (3)
C4—N1	1.459 (5)	O4—K1^ii^	2.818 (4)
C4—C6	1.522 (6)	O4—K1^iii^	2.865 (4)
C4—C5	1.542 (5)	O4—H4A	0.8400
C4—H4	1.0000	K1—O2^iv^	2.773 (3)
C5—O2	1.252 (5)	K1—O2^v^	2.799 (3)
C5—O3	1.256 (5)	K1—O4^v^	2.818 (4)
C6—C7	1.325 (6)	K1—O4^vi^	2.865 (4)
C6—O5	1.398 (4)	K1—C7^v^	3.198 (4)
C7—C8	1.485 (6)		
			
O1—C1—N1	130.1 (4)	C1—N1—C4	122.4 (4)
O1—C1—C2	136.7 (4)	C1—N1—C3	91.1 (3)
N1—C1—C2	93.2 (3)	C4—N1—C3	109.9 (3)
C1—C2—C3	84.5 (3)	O2^iv^—K1—O3	82.78 (10)
C1—C2—H2A	114.6	O2^iv^—K1—O2^v^	79.62 (9)
C3—C2—H2A	114.6	O3—K1—O2^v^	116.68 (9)
C1—C2—H2B	114.6	O2^iv^—K1—O4^v^	176.73 (10)
C3—C2—H2B	114.6	O3—K1—O4^v^	95.70 (10)
H2A—C2—H2B	111.7	O2^v^—K1—O4^v^	103.64 (9)
O5—C3—N1	105.3 (3)	O2^iv^—K1—O2	101.77 (9)
O5—C3—C2	114.9 (4)	O3—K1—O2	46.69 (9)
N1—C3—C2	89.7 (3)	O2^v^—K1—O2	78.70 (9)
O5—C3—H3	114.7	O4^v^—K1—O2	79.11 (9)
N1—C3—H3	114.7	O2^iv^—K1—O4^vi^	79.21 (9)
C2—C3—H3	114.7	O3—K1—O4^vi^	130.76 (10)
N1—C4—C6	102.0 (3)	O2^v^—K1—O4^vi^	104.54 (9)
N1—C4—C5	116.2 (3)	O4^v^—K1—O4^vi^	99.74 (9)
C6—C4—C5	115.9 (3)	O2—K1—O4^vi^	176.76 (10)
N1—C4—H4	107.4	O2^iv^—K1—C5	90.29 (9)
C6—C4—H4	107.4	O3—K1—C5	23.47 (10)
C5—C4—H4	107.4	O2^v^—K1—C5	96.56 (11)
O2—C5—O3	125.0 (3)	O4^v^—K1—C5	89.41 (10)
O2—C5—C4	117.7 (4)	O2—K1—C5	23.45 (9)
O3—C5—C4	117.2 (3)	O4^vi^—K1—C5	154.16 (10)
O2—C5—K1	64.0 (2)	O2^iv^—K1—C7^v^	137.98 (11)
O3—C5—K1	62.1 (2)	O3—K1—C7^v^	124.63 (12)
C4—C5—K1	171.2 (3)	O2^v^—K1—C7^v^	60.23 (10)
O2—C5—K1^i^	44.8 (2)	O4^v^—K1—C7^v^	45.13 (10)
O3—C5—K1^i^	115.7 (3)	O2—K1—C7^v^	83.12 (10)
C4—C5—K1^i^	106.1 (2)	O4^vi^—K1—C7^v^	98.25 (10)
K1—C5—K1^i^	81.24 (8)	C5—K1—C7^v^	105.17 (11)
C7—C6—O5	121.1 (4)	O2^iv^—K1—C8^vi^	89.53 (10)
C7—C6—C4	128.8 (4)	O3—K1—C8^vi^	154.09 (11)
O5—C6—C4	110.0 (3)	O2^v^—K1—C8^vi^	85.89 (9)
C6—C7—C8	127.1 (4)	O4^v^—K1—C8^vi^	90.62 (10)
C6—C7—K1^ii^	105.7 (3)	O2—K1—C8^vi^	158.79 (11)
C8—C7—K1^ii^	90.4 (2)	O4^vi^—K1—C8^vi^	23.49 (9)
C6—C7—H7	116.5	C5—K1—C8^vi^	177.48 (12)
C8—C7—H7	116.5	C7^v^—K1—C8^vi^	76.57 (11)
K1^ii^—C7—H7	71.9	O2^iv^—K1—C5^iv^	18.57 (9)
O4—C8—C7	106.4 (3)	O3—K1—C5^iv^	68.43 (10)
O4—C8—K1^iii^	52.9 (2)	O2^v^—K1—C5^iv^	96.76 (10)
C7—C8—K1^iii^	86.5 (2)	O4^v^—K1—C5^iv^	158.42 (10)
O4—C8—K1^ii^	49.2 (2)	O2—K1—C5^iv^	98.32 (9)
C7—C8—K1^ii^	64.7 (2)	O4^vi^—K1—C5^iv^	81.62 (9)
K1^iii^—C8—K1^ii^	76.39 (8)	C5—K1—C5^iv^	81.24 (8)
O4—C8—H8A	110.5	C7^v^—K1—C5^iv^	156.37 (11)
C7—C8—H8A	110.5	C8^vi^—K1—C5^iv^	97.88 (10)
K1^iii^—C8—H8A	160.2	O2^iv^—K1—C8^v^	159.90 (10)
K1^ii^—C8—H8A	101.1	O3—K1—C8^v^	115.98 (12)
O4—C8—H8B	110.5	O2^v^—K1—C8^v^	85.05 (9)
C7—C8—H8B	110.5	O4^v^—K1—C8^v^	22.62 (9)
K1^iii^—C8—H8B	72.8	O2—K1—C8^v^	87.75 (10)
K1^ii^—C8—H8B	149.1	O4^vi^—K1—C8^v^	92.28 (10)
H8A—C8—H8B	108.6	C5—K1—C8^v^	104.40 (10)
C5—O2—K1^i^	116.6 (3)	C7^v^—K1—C8^v^	24.83 (10)
C5—O2—K1^ii^	136.3 (3)	C8^vi^—K1—C8^v^	76.39 (8)
K1^i^—O2—K1^ii^	101.51 (10)	C5^iv^—K1—C8^v^	173.89 (10)
C5—O2—K1	92.6 (2)	O2^iv^—K1—K1^ii^	90.24 (7)
K1^i^—O2—K1	101.77 (9)	O3—K1—K1^ii^	81.25 (7)
K1^ii^—O2—K1	100.16 (10)	O2^v^—K1—K1^ii^	39.02 (7)
C5—O3—K1	94.5 (2)	O4^v^—K1—K1^ii^	92.38 (7)
C8—O4—K1^ii^	108.2 (2)	O2—K1—K1^ii^	39.68 (6)
C8—O4—K1^iii^	103.6 (3)	O4^vi^—K1—K1^ii^	143.56 (7)
K1^ii^—O4—K1^iii^	99.74 (9)	C5—K1—K1^ii^	58.85 (8)
C8—O4—H4A	109.5	C7^v^—K1—K1^ii^	66.94 (8)
K1^ii^—O4—H4A	133.2	C8^vi^—K1—K1^ii^	123.66 (8)
K1^iii^—O4—H4A	97.3	C5^iv^—K1—K1^ii^	99.19 (7)
C6—O5—C3	109.4 (3)	C8^v^—K1—K1^ii^	85.90 (8)
			
O1—C1—C2—C3	168.2 (6)	C5—O2—K1—O3	−6.0 (2)
N1—C1—C2—C3	−9.2 (3)	K1^i^—O2—K1—O3	111.90 (16)
C1—C2—C3—O5	−98.0 (4)	K1^ii^—O2—K1—O3	−143.96 (16)
C1—C2—C3—N1	8.8 (3)	C5—O2—K1—O2^v^	138.9 (2)
N1—C4—C5—O2	151.2 (4)	K1^i^—O2—K1—O2^v^	−103.25 (11)
C6—C4—C5—O2	31.4 (5)	K1^ii^—O2—K1—O2^v^	0.89 (9)
N1—C4—C5—O3	−30.8 (6)	C5—O2—K1—O4^v^	−114.7 (3)
C6—C4—C5—O3	−150.6 (4)	K1^i^—O2—K1—O4^v^	3.21 (10)
N1—C4—C5—K1	52 (2)	K1^ii^—O2—K1—O4^v^	107.35 (10)
C6—C4—C5—K1	−67.5 (19)	C5—O2—K1—O4^vi^	−45.1 (17)
N1—C4—C5—K1^i^	−161.8 (3)	K1^i^—O2—K1—O4^vi^	72.8 (16)
C6—C4—C5—K1^i^	78.4 (4)	K1^ii^—O2—K1—O4^vi^	177 (45)
N1—C4—C6—C7	174.2 (4)	K1^i^—O2—K1—C5	117.9 (3)
C5—C4—C6—C7	−58.6 (6)	K1^ii^—O2—K1—C5	−138.0 (3)
N1—C4—C6—O5	−2.6 (4)	C5—O2—K1—C7^v^	−160.2 (3)
C5—C4—C6—O5	124.7 (4)	K1^i^—O2—K1—C7^v^	−42.33 (11)
O5—C6—C7—C8	−3.7 (7)	K1^ii^—O2—K1—C7^v^	61.82 (11)
C4—C6—C7—C8	179.9 (4)	C5—O2—K1—C8^vi^	−176.9 (3)
O5—C6—C7—K1^ii^	−106.5 (4)	K1^i^—O2—K1—C8^vi^	−59.1 (3)
C4—C6—C7—K1^ii^	77.1 (5)	K1^ii^—O2—K1—C8^vi^	45.1 (3)
C6—C7—C8—O4	−137.0 (5)	C5—O2—K1—C5^iv^	43.6 (2)
K1^ii^—C7—C8—O4	−26.9 (3)	K1^i^—O2—K1—C5^iv^	161.47 (10)
C6—C7—C8—K1^iii^	173.3 (5)	K1^ii^—O2—K1—C5^iv^	−94.39 (11)
K1^ii^—C7—C8—K1^iii^	−76.57 (10)	C5—O2—K1—C8^v^	−135.7 (3)
C6—C7—C8—K1^ii^	−110.1 (5)	K1^i^—O2—K1—C8^v^	−17.85 (11)
O3—C5—O2—K1^i^	−92.7 (4)	K1^ii^—O2—K1—C8^v^	86.29 (10)
C4—C5—O2—K1^i^	85.1 (4)	C5—O2—K1—K1^ii^	138.0 (3)
K1—C5—O2—K1^i^	−104.54 (19)	K1^i^—O2—K1—K1^ii^	−104.15 (11)
O3—C5—O2—K1^ii^	119.4 (4)	O2—C5—K1—O2^iv^	−120.1 (3)
C4—C5—O2—K1^ii^	−62.7 (5)	O3—C5—K1—O2^iv^	70.9 (3)
K1—C5—O2—K1^ii^	107.6 (3)	C4—C5—K1—O2^iv^	−16.8 (18)
K1^i^—C5—O2—K1^ii^	−147.8 (5)	K1^i^—C5—K1—O2^iv^	−163.73 (9)
O3—C5—O2—K1	11.8 (5)	O2—C5—K1—O3	169.1 (4)
C4—C5—O2—K1	−170.3 (3)	C4—C5—K1—O3	−87.6 (18)
K1^i^—C5—O2—K1	104.54 (19)	K1^i^—C5—K1—O3	125.4 (3)
O2—C5—O3—K1	−12.0 (5)	O2—C5—K1—O2^v^	−40.5 (2)
C4—C5—O3—K1	170.1 (3)	O3—C5—K1—O2^v^	150.5 (3)
K1^i^—C5—O3—K1	−63.5 (2)	C4—C5—K1—O2^v^	62.8 (18)
C7—C8—O4—K1^ii^	32.7 (4)	K1^i^—C5—K1—O2^v^	−84.14 (9)
K1^iii^—C8—O4—K1^ii^	105.23 (18)	O2—C5—K1—O4^v^	63.2 (2)
C7—C8—O4—K1^iii^	−72.5 (3)	O3—C5—K1—O4^v^	−105.9 (3)
K1^ii^—C8—O4—K1^iii^	−105.23 (18)	C4—C5—K1—O4^v^	166.5 (18)
C7—C6—O5—C3	173.7 (4)	K1^i^—C5—K1—O4^v^	19.52 (9)
C4—C6—O5—C3	−9.2 (4)	O3—C5—K1—O2	−169.1 (4)
N1—C3—O5—C6	16.9 (4)	C4—C5—K1—O2	103.3 (19)
C2—C3—O5—C6	113.9 (4)	K1^i^—C5—K1—O2	−43.7 (2)
O1—C1—N1—C4	−53.6 (7)	O2—C5—K1—O4^vi^	174.7 (2)
C2—C1—N1—C4	124.1 (4)	O3—C5—K1—O4^vi^	5.7 (4)
O1—C1—N1—C3	−168.1 (6)	C4—C5—K1—O4^vi^	−82.0 (19)
C2—C1—N1—C3	9.6 (3)	K1^i^—C5—K1—O4^vi^	131.1 (2)
C6—C4—N1—C1	−91.7 (5)	O2—C5—K1—C7^v^	20.4 (3)
C5—C4—N1—C1	141.3 (4)	O3—C5—K1—C7^v^	−148.7 (3)
C6—C4—N1—C3	13.0 (4)	C4—C5—K1—C7^v^	123.7 (18)
C5—C4—N1—C3	−114.0 (4)	K1^i^—C5—K1—C7^v^	−23.27 (12)
O5—C3—N1—C1	106.3 (3)	O2—C5—K1—C8^vi^	154 (2)
C2—C3—N1—C1	−9.5 (3)	O3—C5—K1—C8^vi^	−15 (2)
O5—C3—N1—C4	−18.9 (4)	C4—C5—K1—C8^vi^	−103 (3)
C2—C3—N1—C4	−134.7 (3)	K1^i^—C5—K1—C8^vi^	110 (2)
C5—O3—K1—O2^iv^	−107.7 (3)	O2—C5—K1—C5^iv^	−136.3 (2)
C5—O3—K1—O2^v^	−33.2 (3)	O3—C5—K1—C5^iv^	54.6 (3)
C5—O3—K1—O4^v^	75.2 (3)	C4—C5—K1—C5^iv^	−33.0 (19)
C5—O3—K1—O2	6.0 (2)	K1^i^—C5—K1—C5^iv^	180.0
C5—O3—K1—O4^vi^	−176.7 (2)	O2—C5—K1—C8^v^	46.1 (3)
C5—O3—K1—C7^v^	37.6 (3)	O3—C5—K1—C8^v^	−123.0 (3)
C5—O3—K1—C8^vi^	178.5 (2)	C4—C5—K1—C8^v^	149.4 (18)
C5—O3—K1—C5^iv^	−120.0 (3)	K1^i^—C5—K1—C8^v^	2.41 (12)
C5—O3—K1—C8^v^	64.7 (3)	O2—C5—K1—K1^ii^	−30.0 (2)
C5—O3—K1—K1^ii^	−16.4 (2)	O3—C5—K1—K1^ii^	161.0 (3)
C5—O2—K1—O2^iv^	62.1 (3)	C4—C5—K1—K1^ii^	73.4 (18)
K1^i^—O2—K1—O2^iv^	180.0	K1^i^—C5—K1—K1^ii^	−73.62 (7)
K1^ii^—O2—K1—O2^iv^	−75.85 (11)		

Symmetry codes: (i) *x*+1, *y*, *z*; (ii) *x*+1/2, −*y*+3/2, −*z*+2; (iii) *x*+3/2, −*y*+3/2, −*z*+2; (iv) *x*−1, *y*, *z*; (v) *x*−1/2, −*y*+3/2, −*z*+2; (vi) *x*−3/2, −*y*+3/2, −*z*+2.

### Hydrogen-bond geometry (Å, °)

**Table:**

*D*—H···*A*	*D*—H	H···*A*	*D*···*A*	*D*—H···*A*
O4—H4A···O3^vii^	0.84	1.90	2.673 (5)	153

Symmetry codes: (vii) *x*+1, *y*+1, *z*.
